# The histone deacetylase inhibitor belinostat (PXD101) suppresses bladder cancer cell growth in vitro and in vivo

**DOI:** 10.1186/1479-5876-5-49

**Published:** 2007-10-12

**Authors:** Michael T Buckley, Joanne Yoon, Herman Yee, Luis Chiriboga, Leonard Liebes, Gulshan Ara, Xiaozhong Qian, Dean F Bajorin, Tung-Tien Sun, Xue-Ru Wu, Iman Osman

**Affiliations:** 1 Urology, New York University School of Medicine, New York, USA; 2 Pathology, New York University School of Medicine, New York, USA; 3 Medicine, New York University School of Medicine, New York, USA; 4 Veterans Affairs Medical Center, New York, USA; 5 Medicine, Memorial Sloan-Kettering Cancer Center, New York, USA; 6 Curagen Corporation, Branford, USA

## Abstract

**Background:**

Treatment options for patients with recurrent superficial bladder cancer are limited, necessitating aggressive exploration of new treatment strategies that effectively prevent recurrence and progression to invasive disease. We assessed the effects of belinostat (previously PXD101), a novel histone deacetylase inhibitor, on a panel of human bladder cancer cell lines representing superficial and invasive disease, and on a transgenic mouse model of superficial bladder cancer.

**Methods:**

Growth inhibition and cell cycle distribution effect of belinostat on 5637, T24, J82, and RT4 urothelial lines were assessed. Ha-*ras *transgenic mice with established superficial bladder cancer were randomized to receive either belinostat or vehicle alone, and assessed for bladder weight, hematuria, gene expression profiling, and immunohistochemistry (IHC).

**Results:**

Belinostat had a significant linear dose-dependent growth inhibition on all cell lines (IC_50 _range of 1.0–10.0 μM). The 5637 cell line, which was derived from a superficial papillary tumor, was the most sensitive to treatment. Belinostat (100 mg/kg, intraperitoneal, 5 days each week for 3 weeks) treated mice had less bladder weight (p < 0.05), and no hematuria compared with 6/10 control mice that developed at least one episode. IHC of bladder tumors showed less cell proliferation and a higher expression of p21^WAF1 ^in the belinostat-treated mice. Gene expression profile analysis revealed 56 genes significantly different in the treated group; these included the upregulation of p21^WAF1^, induction of core histone deacetylase (HDAC), and cell communication genes.

**Conclusion:**

Our data demonstrate that belinostat inhibits bladder cancer and supports the clinical evaluation of belinostat for the treatment of patients with superficial bladder cancer.

## Background

Bladder cancer is a major health care problem in the United States and accounts for approximately 13,000 deaths annually [1]. The majority of bladder tumors are initially diagnosed as superficial, however, 70% of patients experience recurrence, and 30% progress to invasive disease [2]. This high rate of recurrence requires patients to undergo lifelong follow-up exams, prophylactic treatments, and additional surgical resection. This protracted natural prevalence of bladder cancer is estimated to affect approximately 500,000 people, and the management of this disease exceeds $4 billion in healthcare expenditures annually [2,3]. It is critically important to aggressively explore pharmacological treatment strategies that can effectively prevent superficial bladder cancer recurrence and progression to invasive disease.

Histone deacetylase inhibitors (HDACIs) represent a new mechanistic class of anti-cancer therapeutics that target HDAC enzymes and have been shown to: arrest growth of cancer cells (including drug resistant subtypes), induce apoptosis, promote differentiation, inhibit angiogenesis, and sensitize cancer cells to overcome drug resistance when used in combination with other anti-cancer agents. Although many HDACIs have been shown to enhance histone acetylation and to increase the expression of tumor suppressor genes in cancerous cells, the exact mechanism(s) that HDACIs effectively inhibit cancer cell growth remains an area of active investigation, and may involve the acetylation of both histone and nonhistone proteins.

HDACIs represent a promising new class of antineoplastic agents for the treatment of bladder cancer. A Phase I clinical trial of suberoylanilide hydroxamic acid (SAHA) showed that 2 out of 4 bladder cancer patients responded to treatment with objective tumor regression and clinical improvement [4]. A new hydroxamate type HDACI known as belinostat was chosen for this study because in vitro experiments showed that it had a potent anti-tumor effect at sub- to low micromolar IC_50 _potency in several tumor cell lines [5-8]. Phase I clinical studies have also suggested that belinostat and other HDACIs have anti-tumor effects [9-12], and that belinostat can specifically inhibit tumor growth in animal models at non-toxic concentrations [5,6,8]. We have examined the effects of PXD101 on bladder tumor cell growth and proliferation, both in vitro and in vivo.

Because the majority of bladder cancer is initially diagnosed as superficial and frequently progresses to invasive disease, we chose to use an expanded panel of human transitional cell carcinoma (TCC) cell lines to include superficial variants in addition to the more commonly used highly invasive disease variants.

The lack of a functionally relevant model system for in vivo testing of potential agents has also limited bladder cancer research and therapy development. Currently, anticancer agents are screened in vivo using human xenograft tumor models grown subcutaneously in athymic mice before initiation of a clinical trial. In many cases, xenografts are selected to suit the putative mechanism of the agent tested, the approach being one of proof of principal in an in vivo model, rather than testing the new agent in a clinically relevant and predictive model. Our group has developed a transgenic mouse model of bladder tumorigenesis using a urothelium-specific promoter to drive the urothelial expression of specific activated tumor oncogenes [13-15]. One of these models expressed, in a urothelium-specific manner, a constitutively active Ha-*ras*, known to be a frequent event in about 30–40% of human bladder cancers [16,17]. Homozygous mice harboring two alleles of the Ha-*ras *mutant consistently developed low-grade, non-invasive, superficial papillary bladder tumors. These transgenic mice have been characterized in detail and were chosen for our in vivo studies. [14-16,18,19]. Ha-*ras *mice reproducibly develop superficial bladder cancer by 3 months of age and continue to form low-grade superficial papillary tumors that rapidly increase in size in the following 3 months. These mice eventually succumb to obstructive neuropathy at 6–7 months. This reproducible and predictable time course of tumor onset and development lent itself as a well-defined model for screening belinostat and other potential chemotherapeutic agents to test their abilities to hinder the development and progression of superficial bladder cancer.

Herein, we show that belinostat treatment inhibited cell growth and proliferation in a dose-dependent fashion and caused cell cycle arrest in our panel of urinary bladder cancer cell lines. We also show that treatment of Ha-*ras *transgenic bladder cancer mice with belinostat decreased bladder tumor growth with no apparent toxicity and induced p21^WAF1 ^and other HDAC core and cell communication genes. These findings suggest that belinostat may represent a novel adjuvant treatment for patients with superficial recurrent bladder cancer.

## Methods

### Cell culture, proliferation assay and belinostat

The human urinary bladder carcinoma cell lines 5637, T24, J82 and RT4 were obtained from the American Type Culture Collection (Manassas, VA). All tumor cell lines were maintained in DMEM (Sigma; St Louis, MO), supplemented with 10% FBS, and maintained at 37°C with 5% CO_2_. Cells were seeded into 96-well tissue culture plates (J82 at 2000 cells/well; 5637, T24 and RT4 at 4000 cells/well), allowed to attach and grow for 24 h, exposed to 1–10 μM of belinostat for 48 h, and cell proliferation was assessed using the WST-1 tetrazolium salt cleavage assay kit (Chemicon; Temecula, CA) as per the manufacturer's instructions.

Belinostat has been previously described [6] and was prepared as a 10 mM stock in DMSO/PBS for in vitro studies. For animal studies, belinostat was dissolved in L-Arginine to give a final concentration of 20 mg/ml. This formulation gave sufficient solubility for doses of ≤ 40 mg/kg. Belinostat was kindly provided by CuraGen Corp., TopoTarget and the National Cancer Institute.

### Cell cycle analysis

FACS analysis was performed on cells treated with 5 μM belinostat for 48 h, harvested with trypsin-EDTA (Sigma; St Louis, MO), and fixed in absolute ethanol overnight at -20°C. Immediately before analysis, cells were treated with 200 ug/mL DNAse-free RNAseA (Sigma; St Louis, MO) for 30 minutes at 37°C, then treated with 1 mg/mL propidium iodide (Sigma; St Louis, MO). Cells were analyzed using a FACScan (Becton Dickinson; Franklin Lakes, NJ) at an excitation wavelength of 488 nm at the NYU Cancer Institute's Flow Cytometry and Cell Sorting Core Facility.

### Generation of *UPII*-Ha-*ras *transgenic mice and belinostat treatment

The transgenic model used for this study specifically expressed a constitutively activated Ha-*ras *oncogene in the urothelium under the control of a 30-kb mouse uroplakin II promoter [15]. Intercrossing of heterozygous mice yielded homozygous offspring that consistently and reproducibly developed superficial bladder cancers at well-defined time points [19,20]. Homozygous mice were distinguished from heterozygotes by Southern blotting of tail genomic DNA. DNA was digested with *Nco*I, resolved by gel electrophoresis, and hybridized with a ^32^P-labeled, UPII probe (600-base pairs), which allowed detection of both the endogenous UPII gene and the mUPII/Ha-*ras*-M transgene. Densitometric analysis of the genomic Southern blot was used to calculate the relative amount of transgene present by comparing transgene with endogenous UPII gene. Breeding and housing of mice were conducted at the Manhattan VA Medical Center under the guidance of Tung-Tien Sun and Xue-Ru Wu. Animal Studies were carried out at the Manhattan VA Medical Center under IACUC guidelines of the New York Harbor Healthcare System and conformed to their guidelines for the welfare of animals in experimental neoplasia. The starting point of belinostat was set at 3 months of age when all homozygous mice were known to have established bladder tumors. Twenty Ha-*ras *mice were randomized into two groups of 10 per group. Ten mice received intraperitoneal (IP) injections containing belinostat dissolved in L-Arginine each day for 5 days each week for 3 weeks (100 mg/kg, IP, 5 days each week for 3 weeks), and 10 received IP injections with L-Arginine alone following the same dose scheduling. Mice were weighed twice weekly, checked daily for gross hematuria by applying light pressure on the bladder, and monitored for any changes in behavior or condition. One day after the last dosing (when mice were 3 months and 22 days old) all twenty mice were sacrificed, bladders were removed, weighed after voiding of all urine, necroscopied, divided for RNA isolation, and paraffin embedded for IHC.

### Histopathology of mouse bladder tumors

All bladders and tumors were analyzed histopathologically and all were confirmed to be superficial with no evidence of invasion. We also looked for differences in necrosis, mitotic figures, and the extent of tumor burden present in all bladders.

### Microarray Analysis

All mouse bladders were processed for total RNA isolation and all subsequent technical procedures including purity and concentration of RNA, cDNA synthesis, biotin labeling of cRNA, and hybridization and scanning of arrays were performed by Genome Explorations, Inc. (Memphis, TN). Briefly, RNA integrity was determined by capillary electrophoresis using the RNA 6000 Nano Lab-on-a-Chip kit and the Bioanalyzer 2100 (Agilent Technologies). In order to obtain sufficient highly pure RNA for gene profiling it was essential to identify and pool the best quality RNA from three animal bladders per treatment group (15 μg). Our transgenic mice represented a homogeneous biologic entity. Similarly, other investigators using the same GeneChips have pooled RNA from transgenic mice organs for subsequent microarray analysis [21,22]. Preparation of the cRNA and the subsequent microarray processes were performed as described in the Affymetrix GeneChip expression analysis technical manual (Affymetrix; Santa Clara, CA). Briefly, cRNA was hybridized to Affymetrix MOE 430 2.0 short oligomer arrays, which detect approximately 45,000 mouse transcripts representing over 34,000 well-characterized mouse genes. The results were analyzed using programs resident in GeneChip Operating System v1.4 (GCOS; Affymetrix). Conversion of gene names or accession numbers to Affymetrix probe set IDs was accomplished using NetAffx. Probe sets were identified by pair-wise comparison in GCOS using a 2-fold change threshold, and the GCOS-generated Change calls and Detection calls were used in our filtering criteria to identify robust expression changes. Signal intensity heat map figures were generated using . Due to an inadequate amount of bladder tissue, gene analysis was performed on pooled RNA samples with no replicates. Our gene analysis was an investigational type of array given that a traditional p-value could not be generated due to the lack of sufficient individual RNA samples.

### Immunohistochemistry of mouse bladder tumors

Freshly dissected bladder tissues were fixed in 10% buffered formalin and processed routinely for paraffin embedding. Three-micron tissue sections were stained with hemotoxylin/eosin and examined microscopically. To determine the proliferative and apoptotic capacity of the tumors, we stained sections for the expression of proliferation specific antigen (Ki-67) using the mouse monoclonal antibody MIB1 (Immunotech SA; France), and assessed the expression of p21^WAF1 ^using MAb clone 2G12 (PharMingen; San Diego, CA), both as described previously [23].

### Image quantitation of Ki67 and p21^WAF1 ^IHC staining

The quantitative digital analysis of the IHC stained slides for Ki67 and p21^WAF1 ^involved the following modifications from methodology previously developed [24,25] using Kodak Molecular Imaging (MI) software (Ver 4.0, New Haven, CT): all slides were reviewed by a pathologist who captured a representative area using Olympus Digital Vision v3.0 (Center Valley, PA) at 20× objective magnification and output as a TIFF file. The image was imported into Adobe Photoshop CS2 (Adobe Systems Inc.; San Jose CA) and the image color was standardized across all images using the auto level function. In Photoshop, the wand function was then used to subtract immunonegative portions of the image. Tumor images excluded areas containing preparation artifact and any necrotic or benign regions. The final image was imported into Kodak MI where automatic conversion to grayscale occurred followed by utilization of the "automatic region-of-interest" function for the entire image. The density slice mode was used with the threshold visually adjusted (over the setting of 0–255) to select for only immunopositive staining tumor pixels. The pixel size was unrestricted, and the automatic find function was set to search for immunopositive pixels using smooth edges. The interior area of the positively staining pixel regions-of-interest was determined by the Kodak MI analysis, and the sum was calculated using Microsoft Excel. To obtain percent staining, the sum of the interior area of the positively staining pixels was divided by the entire interior pixel area for the image being analyzed. To obtain fold change in staining for p21^WAF1 ^in the belinostat-treated mice over the arginine-treated group, the percent staining of the belinostat group was divided by the percent staining of the arginine treatment group. To obtain fold change in staining for Ki67 in the arginine treated mice over the belinostat-treated group, the percent staining of the arginine group was divided by the percent staining of the belinostat treated group.

### Statistical Analysis

Cell proliferation and FACS analysis experiments were performed at least three times independently, with 3–8 repeats at each data point. Statistical analysis was performed using GraphPad Instat version 3.0 (Graph Pad Software Inc.; San Diego, CA). Statistical significance was calculated using the Students two-tailed t test, where p < 0.05 was considered significant.

## Results

### Belinostat inhibited bladder cancer cell growth

**Figure 1:**
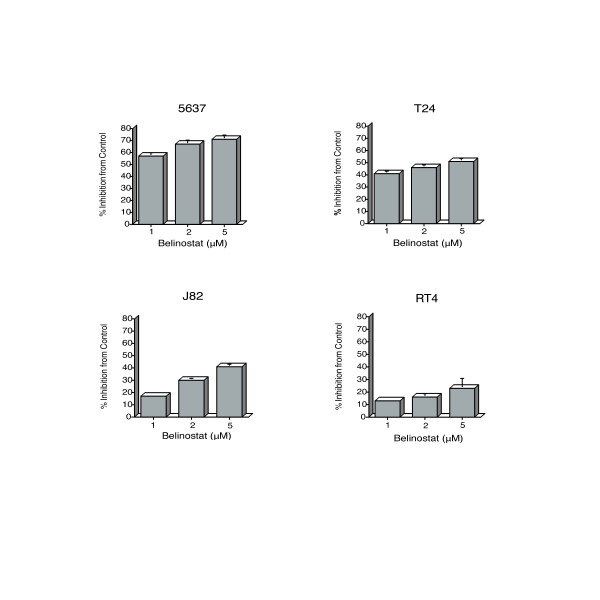
Inhibition of bladder cancer cell proliferation by belinostat at 1, 2 and 5 μM for 48 h in the human urinary bladder cancer cell lines 5637, T24, J82 and RT4. Percent inhibition from control was determined using the WST-1 tetrazolium salt cleavage assay. Bars are representative of at least 3 independent experiments and are the mean of at least 8 wells per condition. Error bars indicate SEM.

The in vitro treatment of all four urothelial carcinoma cell lines at 1–5 μM belinostat for 48 h caused a dose-dependent inhibition of proliferation, with the most potent inhibitory effect occurring on 5637 cells (IC50 of 1.0 μM), and the least effect occurring on RT4 cells (IC50 of 10.0 μM). T24 and J82 cell lines had an IC50 of 3.5 and 6.0 μM, respectively. Treatment with 5 μM belinostat for 48 h caused a 71% (± 0.2, SEM) decrease in cell growth and proliferation for 5637 cells, 51% (± 1.0) for T24, 41% (± 2.0) for J82, and 23% (± 7.9) for RT4 cells (Figure 1). All cell lines, except the RT4 line, showed a significant growth inhibition (GI) when compared to control at all concentrations of belinostat (1, 2 and 5 μM) (p < 0.001). RT4 cells only showed a significant GI at 5 μM belinostat when compared to control (p = 0.01).

### Induction of cell cycle arrest by belinostat

**Figure 2:**
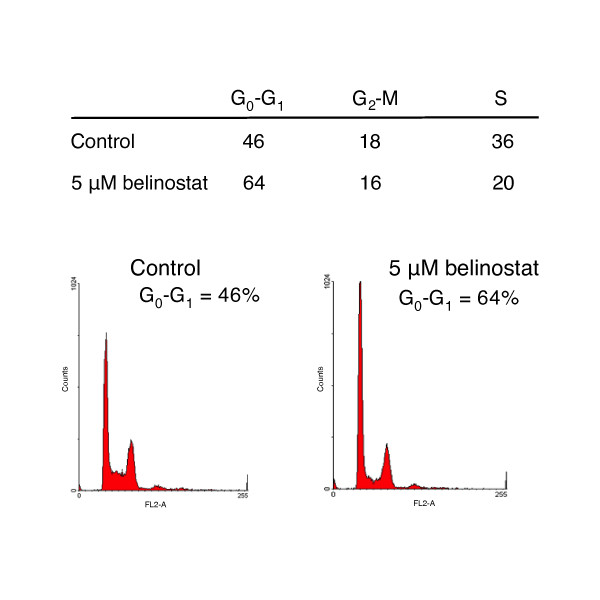
Effect of belinostat on cell cycle distribution in 5637 cells. 5637 cells were the most sensitive to belinostat treatment and showed the greatest accumulation of G_0_-G_1 _cells, decrease in S phase cells and increase in G_2_-M phase cells after treatment. Cells cultured with 5 μM belinostat for 48 h were analyzed by flow cytometry, and percent cell cycle distribution was assessed by standard histogram analysis.

Cell cycle analysis showed that, 48 h after the 5637 bladder carcinoma cells were treated with 5 μM belinostat, there was an 18% (± 1.0, SD) increase of cells in the G_0_-G_1 _phase, and a 16% (± 1.0) decrease in S phase (Figure 2); indicating the cells were arrested at the G_0_-G_1_transition. The J82 cells showed a moderate 10% (± 0.7) decrease in S phase cells. RT4 cells showed minor changes in cell cycle parameters: 6% (± 0.8) build up of cells in G_0_-G_1_, and 5% (± 0.4) decrease in S phase.

### Belinostat reduced mice bladder weights, decreased hematuria and was well-tolerated

**Figure 3:**
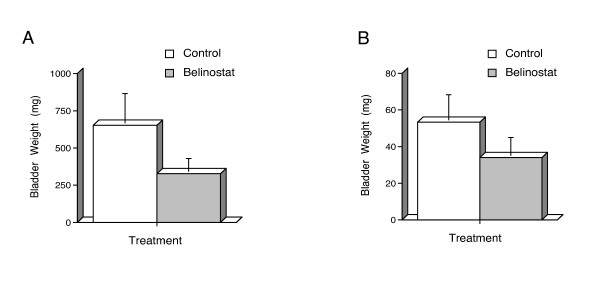
Belinostat decreases both male and female bladder weights in transgenic superficial bladder cancer mice. Ha-*ras *transgenic mice with established superficial bladder cancer were randomized to receive either belinostat dissolved in L-Arginine (100 mg/kg, IP, qd, 5 days on, 2 days off, 3 cycles) or L-Arginine alone as a control following the same dose scheduling. All bladders were voided of urine prior to weighing. Normal non-transgenic mouse bladders weigh approximately 10.0 mg at the same age, and Ha-*ras *transgenic mice with superficial bladder cancer have a 3 fold and higher bladder weight. **A**, Male belinostat-treated mice (n = 4, 327.5 mg average weight) showed a two-fold decrease (50%, p = 0.03) in bladder weight versus control (n = 4, 652.5 mg average weight). **B**, Female belinostat-treated mice (n = 5, 34.0 mg average weight) showed a 36% decrease (p = 0.04) in bladder weight versus control (n = 6, 53.3 mg average weight).

**Figure 4:**
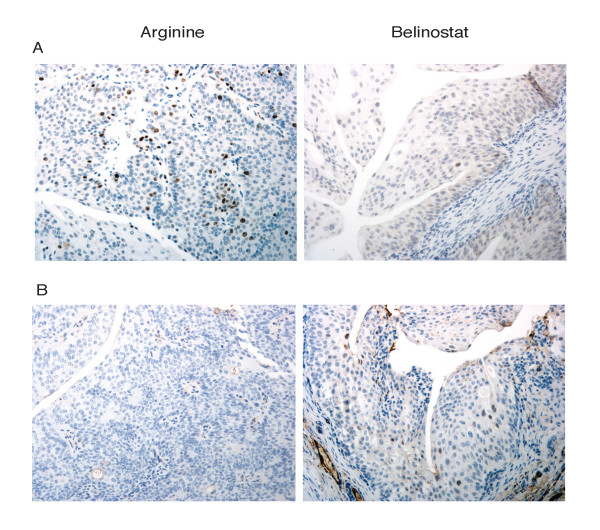
IHC staining of transgenic mice bladders for **A**, the cell proliferation marker Ki67 and **B**, P21 in L-Arginine treated (left panel) and belinostat-treated bladder cancer mice (right panel). Images are representative of 9 belinostat-treated mice and 10 control treated with vehicle alone (L-Arginine) selected by a pathologist. All images at 20× objective magnification.

The transgenic mice used in this study all had established superficial bladder cancer when treatment was initiated, therefore this study was one that explored the effect of belinostat on established superficial bladder cancer, and not one that sought to prevent initiation. The bladder epithelium of our Ras-expressing transgenic mice undergo tumorigenic changes resulting in a 300% increase in bladder weight at 3 months of age (30 mg Ha-*ras *bladder versus 10 mg normal non-transgenic mouse bladder). Consistent with previous studies in non-transgenic mice [26], the increase in male bladder weight due to tumor formation occurred at a faster rate than in females. Belinostat caused a 50% (p = 0.03; Figure 3a) and 36% (p = 0.04; Figure 3b) decrease in the weights of Ras-expressing bladders of the male and female transgenic mice, respectively. While untreated Ras-expressing transgenic mice showed many episodes of hematuria (male mice 2/4: one mouse with one episode on day 3, and one mouse with one episode on day 7 and day 16; female mice 4/6: two mice with one episode on day 7, and two mice with one episode on day 16), none of the belinostat-treated mice had hematuria (male mice 0/4, female mice 0/5). The lack of any incidence of hematuria demonstrated that all mice being treated with belinostat experienced decreased progression of bladder disease compared to vehicle alone. Haematuria in this model might be considered a sign of bladder cancer. Although development of haematuria is not in complete parallel with the development of bladder cancer, haematuria has been consistently reported as the most common symptom of bladder cancer in humans [27]. The comparison of the rate of haematuria in the control arm versus that in the belinostat treated arm was consistent with our suggestion that haematuria in our mouse model mirrors, at least in part, the human counterpart. In addition, belinostat showed no detectable toxicity as evaluated by weight (male and female belinostat-treated mice showed a 1% (p = 0.19) and 11% (p = 0.70) increase in body weight, respectively. Pathological examination at necroscopy also showed no significant abnormalities (including necrosis or mitotic figures) between the two groups. Bladder tumors in the treated mice were smaller and occupied less space of the total bladder capacity. There were no striking histopathological differences between the two treatment groups, however IHC of Ki67 showed an increase in cell proliferation in the control mice over that of belinostat-treated mice (Figure 4a). IHC analysis also showed an increase of p21^WAF1 ^expression in the belinostat-treated mice over that of the control (Figure 4b).

### Belinostat induced p21^WAF1^, HDAC core and cell communication genes

cDNA microarray studies of mouse bladder tumors revealed 22 HDAC core genes that were significantly up- or downregulated due to belinostat treatment (Table 1). These genes are involved in cell cycle regulation, apoptosis and DNA synthesis. The most prominently upregulated genes due to belinostat treatment were metallothionein 1, hepatoma derived growth factor, CTP synthase, fucosidase, and p21^WAF1^. The most dominantly downregulated genes were clusterin, histone H2be, and tubulin alpha 4. We also determined that 34 cell communication genes were differentially expressed due to belinostat treatment (Table 2).

**Table 1:** Belinostat induces P21^WAF1 ^and other HDAC core genes in transgenic mice bladders. Summary of changes in transgenic mice bladder gene expression for belinostat-treated versus control

**Gene Name**	**Gene Symbol**	**Gene Function**	**Fold Change**
Acidic nuclear phosphoprotein 32 family, member B	*Anp32b*	Cell cycle	1.2
Clusterin	*Clu*	Cell death/apoptosis	-2.2
Cyclin-dependent kinase inhibitor 1A	*P21*	Cell cycle regulation	1.5
Cytidine 5'-triphosphate synthase	*Ctps*	DNA synthesis	1.8
Dehydrogenase/reductase (SDR family) member 3	*Dhrs3*	Short chain alchol dehydrogenase	1.3
Dehydrogenase/reductase (SDR family) member 4	*Dhrs4*	Short chain alchol dehydrogenase	-1.4
Dehydrogenase/reductase (SDR family) member 7	*Dhrs7*	Short chain alchol dehydrogenase	1.3
Dehydrogenase/reductase (SDR family) member 8	*Dhrs8*	Short chain alchol dehydrogenase	1.5
Fucosidase, alpha-L-1, tissue	*Fuca1*	Proteoglycan metabolism	1.6
Glutaredoxin 1 (thioltransferase)	*Glrx1*	Glutathione dep DNA synthesis	1.3
Glutaredoxin 2 (thioltransferase)	*Glrx2*	Glutathione dep DNA synthesis	1.2
Hepatoma-derived growth factor	*hdgf*	Transformation related protein	1.3
Hepatoma-derived growth factor	*hdgf*	Transformation related protein	2.9
Histone 2, H2be	*Hist2h2bb*	Nucelear organization	-1.6
Karyopherin (Importin) beta 1	*kpnb1*	Nucelear translocation	-1.6
Karyopherin (Importin) beta 1	*kpnb1*	Nucelear translocation	1.1
Karyopherin (Importin) beta 1	*kpnb1*	Nucelear translocation	1.4
Metalliothionine 1	*Mt1*	Binds heavy metals	4.3
Tubulin, alpha 2	*Tuba2*	Cytoskeletal structure	1.1
Tubulin, alpha 4	*Tuba4*	Cytoskeletal structure	-1.7
Tubulin, alpha 6	*Tuba6*	Cytoskeletal structure	-1.2
Tubulin, alpha 6	*Tuba6*	Cytoskeletal structure	1.2

**Table 2:** Belinostat induces 34 genes involved in cellular communication. Summary of fold changes in transgenic mice bladder gene expression for belinostat-treated versus control

**Gene Name**	**Gene Symbol**	**Gene Function**	**Fold Change**
Adiponectin, C1Q and collagen domain containing	*Adipoq*	Cardiovascular, homeostasis	3.1
Chemokine (C-C motif) ligand 2	*Ccl2*	Chemotaxis, immune response	2.8
Secreted frizzled-related sequence protein 1	*Sfrp1*	Transmemb. receptor activity	2.5
Metallothionein 2	*Mt2*	Binds metal ions	1.8
Gap junction membrane channel protein alpha 1	*Gja1*	Intercellular channel	1.1
Protein phosphatase 2A	*Ppp2r2a*	Intrinsic catalyst activity	1
Early growth response 2	*Egr2*	DNA and metal ion binding	1.2
Metallothionein 1	*Mt1*	Metal ion binding	1.3
BMP-binding endothelial regulator	*Bmper*	Neg regulation of BMP pathway	1
RAB2	*Rab2*	Member Ras oncogene family	1
ADP-ribosylation factor 4	*Arf4*	GTP binding	1
Cell division cycle 42 homolog	*Cdc42*	GTP binding	1
CD47 antigen	*CD47*	Integrin-assoc. signal transducer	1
Tyrosine 3-monooxygenase activation protein	*Ywahz*	Brain signal transduction	1
Integrin beta 1	*Itgb1*	Fibronectin receptor beta	1.1
Integrin linked kinase	*Ilk*	ATP binding, kinase activity	-1
Real guanine nucleotide dissociation stimulator-like 2	*RgI2*		-1.1
Regulator of G-protein signaling 19	*Rgs19*	Neg regulation of signal transd.	-1.1
Reelin	*Reln*	Axon guidance, brain dev.	-1.2
Conserved helix-loop-helix ubiquitous kinase	*Chuk*	Morphogen. of epithelial sheet	-1.1
MKIAA1154 protein			-1.4
Diacylglycerol kinase zeta	*Dgkz*	Protein kinase C activation	-1.1
Phospholipase D2	*Pld2*	Catalytic activity	-1.2
PTK2 protein tyrosine kinase 2	*Ptk2*	Angiogenesis, blood vessel dev.	-1.7
5 days embryo whole body cDNA	*Bmp4*	Bone	-1.2
Deltex 2 homolog	*Dtx2*	Notch signaling pathway	-1.3
G protein-coupled receptor, family C, group 5, memb C	*Gprc5C*	Signal transduction	-1.4
Disheveled, dsh homolog 1	*Dvl1*	Dendrite morphogenesis	-1.2
Angiotensin receptor-like 1	*Agtrl1*	Signal transduction	-1.4
RAB2B	*Rab2b*	ER to golgi and protein transprt.	-1.1
Intersectin 1	*Itsn1*	Endocytosis, intracellular signal	-1.5
Ras and Rab interactor 1	*Rin1*	Endocytosis, intracellular signal	-1.4
Tripartite motif protein 9	*Trim9*	Synaptic vesicle exocytosis	-2.2
Casitas B-lineage lymphoma	*Cbl*	Ca ion binding, ligase activity	-4

## Discussion

This is the first study to demonstrate the low micromolar potency of belinostat in human bladder cancer cells. Although we did not conduct a comparative study and test any other HDACIs alongside belinostat, we feel that a non-direct comparison to other HDACs is important. Our data demonstrated that in comparison with other HDACIs such as valproic acid and sodium butyrate, belinostat had greater potency, required only 3.5 μM to achieve an IC_50 _in T24 cells, and also had a relatively lower micromolar IC_50 _range of 1.0–10.0 μM for the 5637, J82 and RT4 cell lines (IC_50 _data for J82 and RT4 not shown). Other HDACIs, such as valproic acid, have required millimolar concentrations in order to achieve an IC_50 _in the T24 cell line [28-30]. This high concentration of valproic acid resulted in the dose-limiting neurotoxicity observed in the clinical setting [28]. Other groups have had better success using 10–20 μM SAHA to achieve an IC_50 _on T24 cells [31]. Belinostat had a similar effect on cell cycle distribution compared with other HDACIs such as trichostatin A (TSA), sodium butyrate, and SAHA [29,32,33]. All of these agents have been reported to decrease S-phase and G_2_-M phase cells, and increase the accumulation of G_0_-G_1 _phase cells after treatment.

Our study revealed that the 5637 cells were the most sensitive to the effect of belinostat on cell cycle distribution and proliferation. The preferential response of this cell line might be explained by its genetic profile, as well as the mechanism of action that belinostat exerted on it. 5637 cells are p53 mutant, have a p16 deletion, and express p73 in IHC staining [34]. In the future, screening a patient's tumor for these markers may give an indication of potential favorable clinical response to belinostat.

For assessment of apoptosis, both in vitro assays on all four cell lines and in vivo caspase 3 IHC staining of mice bladders did not show any significant difference between the treated and un-treated groups (data not shown). Therefore, we believe that cell cycle arrest via p21 up-regulation, not apoptosis, is the predominant mechanism of tumor inhibition in our current system.

Gene expression analysis of belinostat-treated mice showed increased p21^WAF1 ^gene transcript expression. This finding was validated by IHC analysis, where p21^WAF1 ^expression in belinostat-treated mice was also upregulated in comparison with control mice. IHC image analysis of Ki67 showed a 17.8 fold increase of cell proliferation in the control mice over that of belinostat-treated mice. IHC image analysis of p21^WAF1 ^expression showed an 11.7 fold increase in the belinostat-treated mice. Expression of the cell cycle kinase inhibitor p21 is one of the most commonly induced genes by HDACIs such as TSA, SAHA, and sodium butyrate [31,33,35-37]. Recent studies have shown that belinostat induces p21^WAF1 ^in ovarian, colon, lung, breast, prostate and melanoma cell lines [6]. p21^WAF1 ^is a cyclin-dependent kinase inhibitor that is associated with activities that lead to cell cycle arrest, and apoptosis. Belinostat also upregulated metallothionine 1, another member of the HDAC core gene family, by 4.3 fold. Metallothioneins are a group of cysteine-rich stress response proteins that scavenge reactive oxygen species and heavy metals. Upregulation of metallothionine 1L has also been reported by treatment of T24 cells by three other HDACIs: SAHA, TSA, and MS-27–275 [38], and treatment of mouse lymphosarcoma cells by TSA and depsipeptide [39]. Tubulin alpha 4 was downregulated in belinostat-treated mice and confirmed previously reported data that tubulin is a target of belinostat [8]. Alteration of microtubulin function is commonly exerted by a wide variety of chemotherapeutic agents such as the vinca alkaloids and taxanes, two families of agents that effectively inhibit cell division, proliferation and function. Disruption of tubulin function has been implicated as a critical downstream event for initiating apoptosis in cancer cells [40].

Conversely, our expression profile results showed that some genes such as histone 2, and those known to regulate DNA synthesis (CTP synthase) and apoptosis (clusterin), were oppositely regulated by belinostat compared to other reports that used different HDACIs on bladder and breast carcinoma cells [38]. One possible explanation for this effect by belinostat could be due to the very nature of HDAC inhibition. HDAC inhibition is known to disrupt cell cycle function due to its alteration of chromatin function in carcinoma cells. This undoubtedly causes alterations in normal nuclear processes involved in cell cycle, apoptosis, and proliferation, and subsequently alters normal gene expression patterns. Belinostat could affect these genes differently than other HDACIs while still being able to induce cell cycle arrest, cell growth inhibition, and p21 expression, as we have demonstrated in our data. Our results illustrate the complexity surrounding the regulation of gene transcription that occurs through chromatin remodeling by all HDACIs, including belinostat. Most importantly, gene expression profiling in our transgenic model showed that belinostat induced a common set of core HDAC genes similar to those previously reported in the T24 human bladder cancer cell line treated with different HDACIs [38].

Gene expression analysis also showed that 34 genes involved in cell communication were significantly up or down regulated due to belinostat treatment. HDACIs are known to alter the expression of genes involved in cellular communication and signal transduction [41]. One of the most predominantly upregulated genes was secreted frizzled-related sequence protein 1 (SFRP1). Dysregulation of the SFRP family in human cancers has been correlated with the HDAC inhibitor Trichostatin A [42]. This gene has also been shown to induce apoptosis in MCF7 breast cancer cells [43]. We also found that belinostat induced the dysregulation of Adiponectin (Adipoq). The altered expression of this gene has also been shown to occur with the HDAC inhibitor valproic acid [44].

While the data in this report establish the link between dose-response relationships in both in vitro and in vivo efficacy models, it is important to note that both the in vivo dosing schedule and in vitro concentration ranges chosen for these experiments are achievable in patients. In the current clinical setting, belinostat is dosed at the MTD (1000 mg/m^2^) given intravenously, which results in a C_max _of ~100 μM and AUC_0-*t *_of ~31 μM*hr/mL (unpublished data), treatments are given 5 times per week in a 3 week cycle. Exposure of cells in culture to belinostat concentrations of 1–5 μM over 48 hr in this study is well within the clinical range and this resulted in significant cell growth inhibition and cell cycle arrest. In accordance with the clinical trial, in this study, belinostat, administered in transgenic mice five times per week, showed efficacy at a dose in the lower range of clinical dosing, 100 mg/kg, human equivalent dose of 300 mg/m^2^. Hence, both in vitro and in vivo dosing of belinostat used in this study are within clinically achievable dosing regimens.

Our Ha-*ras *transgenic model of human bladder cancer offered a unique correlation to the onset and progression of human superficial bladder cancer not available in the xenograft system. In these mice, superficial tumors occupied the entire bladder volume at the endpoint of this study making miscrodissection impractical. Since microdissection could not be performed we weighed the entire bladder from each animal and used it as a surrogate marker to assess tumor burden. However, when all mice were sacrificed and underwent pathological dissection and analysis, all bladder tumors in the belinostat-treated mice were smaller and occupied less space of the total bladder capacity than untreated mice. Belinostat-treated mice had a lower incidence of bladder tumors compared to untreated mice based on total bladder weight. This indicates that belinostat was able to decrease the progression of existing established superficial bladder cancer. Of note, the Ha-*ras *mice used in this study all have low-grade superficial bladder tumors starting at 3 months that progress to occupy the entire bladder and force the mice to succumb to obstructive neuropathy at 6–7 months of age. Although the mice in this study were not allowed to succumb to obstructive neuropathy, we anticipate that untreated mice would succumb to obstructive neuropathy quicker than those mice treated with belinostat based on the former's increased endpoint tumor burden. Another alternative to microdissection would be the use of the novel computed tomography system developed to image the urinary tract and tumors in live mice [45]. This technique may offer potential to quantitatively assess tumor size in superficial transgenic mice in future experiments.

Previous phase I trials of the histone deacetylase inhibitors phenylbutyrate [9] and depsipeptide [10] have shown minimal toxicity to patients. A recent phase 1 trial of MS-275, a benzamide derivative with potent HDAC inhibition and antitumor activity in preclinical models, was used in patients with advanced myeloid leukemias and showed no response by classical criteria, but suggested a potentially better clinical outcome if tested in a cohort of patients with less-advanced disease [46]. A phase 2 trial using vorinostat in combination with carboplatin and paclitaxel showed that both dose schedules used were well tolerated, and the study had encouraging anticancer activity in patients with previously untreated non-small cell lung cancer [47].

When used in combination with established chemotherapeutics such as carboplatin and docetaxel, belinostat was found to synergistically inhibit both in vitro and in vivo ovarian cancer cell growth [8]. Belinostat has also been shown to synergize with 5-fluorouracil to inhibit colon cancer cell growth in vitro and in vivo, and demonstrated a strong rationale for the use of belinostat and 5-fluorouracil in combination in the clinic [5]. Currently, belinostat is undergoing investigation for a wide range of solid and hematologic malignancies either as a single-agent, or in combination with other active anti-cancer agents, including 5-FU, carboplatin, paclitaxel, cis-retinoic acid, azacitidine and Velcade (bortezomib) for Injection. Promising results include good tolerance and a broad range of antitumor activity. Intravenous belinostat is currently being evaluated in multiple clinical trials as a potential treatment for multiple myeloma, T- and B-cell lymphomas, AML, mesothelioma, liver, colorectal, ovarian cancers, either alone or in combination with anti-cancer therapies. An oral formulation of belinostat is also being evaluated in a Phase I clinical trial for patients with advanced solid tumors. Given the well tolerability of belinostat, these results indicate that further investigation of belinostat as a bladder cancer treatment, either used alone or in combination with other chemotherapeutics, is well warranted.

## Conclusion

In this study, we showed that belinostat induced growth inhibition and cell cycle arrest in a panel of human TCC urinary bladder cells in vitro at low micromolar concentrations. Belinostat increased gene and IHC expression of p21^WAF1 ^at both mRNA and protein levels, and treatment with belinostat decreased cell growth and proliferation in our transgenic mouse model of superficial bladder cancer at a concentration that was without apparent toxicity to the mice. Taken together, these findings suggest that belinostat is a potent and relatively tolerable agent for the treatment of superficial urinary bladder cancer.

## Competing interests

The author(s) declare that they have no competing interests.

## Authors' contributions

All authors have read and approved the final manuscript. MB carried out the proliferation and FACs analysis studies, participated in interpretation of the data, data analysis, and manuscript writing. JY carried out the transgenic mice data collection and coordinated the gene expression analysis. HY analyzed the histology and immunohistochemistry. LC performed IHC staining and participated in manuscript drafting. LL participated in data analysis and drafting of the manuscript. GA and XQ participated in study design and drafting of the manuscript. DB participated in study design and the drafting of the manuscript. TS and XW participated in study design, interpretation of the data and manuscript writing. IO designed the study, and led the data interpretation and manuscript writing.
